# Lenalidomide and Chronic Lymphocytic Leukemia

**DOI:** 10.1155/2013/932010

**Published:** 2013-09-19

**Authors:** Ana Pilar González-Rodríguez, Angel R. Payer, Andrea Acebes-Huerta, Leticia Huergo-Zapico, Monica Villa-Alvarez, Esther Gonzalez-García, Segundo Gonzalez

**Affiliations:** ^1^Department of Hematology, Hospital Universitario Central de Asturias, C/Celestino Villamil s/n, 33006 Oviedo, Spain; ^2^Department of Functional Biology, IUOPA, Universidad de Oviedo, C/Julián Claveria s/n, 33006 Oviedo, Spain; ^3^Department of Hematology, Hospital de Cabueñes, C/ Cabueñes s/n, 33394 Gijón, Spain

## Abstract

Lenalidomide is an oral immunomodulatory drug used in multiple myeloma and myelodysplastic syndrome and most recently it has shown to be effective in the treatment of various lymphoproliferative disorders such as chronic lymphocytic leukemia (CLL) and non-Hodgkin lymphoma. The mechanism of action of lenalidomide varies depending on the pathology, and in the case of CLL, it appears to primarily act by restoring the damaged mechanisms of tumour immunosurveillance. This review discusses the potential mechanism of action and efficacy of lenalidomide, alone or in combination, in treatment of CLL and its toxic effects such as tumor lysis syndrome (TLS) and tumor flare reaction (TFR), that make its management different from other hematologic malignancies.

## 1. Introduction on Chronic Lymphocytic Leukemia

Chronic lymphocytic leukemia (CLL) is the most common form of leukemia in adults in Western countries. In our population, the crude rate is 8.99 per 100.000 populations per year and the age-adjusted rate is 3.47 per 100.000 populations per year. Overall survival at 5 and 10 years is ranged between 87% and 73% for low-risk patients and 29% and 16% for high risk [1]. In addition, the clinical course of the disease is very heterogeneous; some patients require treatment after a long time, while others rapidly progress with a few months of survival. Patients who relapse frequently show poor prognostic genetic characteristics, such as del (17p) or TP53, that confer them resistance to cytostatic drugs such as fludarabine [2, 3].

 Microenvironment and immune system play a key role in the pathogenesis of CLL. Tissue microenvironment signals promote leukemic cell proliferation, survival, and resistance to chemotherapy. For instance, IL-4 secreted by T cells induces the overexpression of antiapoptotic proteins, such as bcl-2, in leukemic cells [4]. Additionally, one of the key features of CLL is the development of a progressive immunodeficiency which is associated with an increased incidence of infections and secondary malignancies. In addition, numerous qualitative and quantitative alterations affecting all components of the immune system including T cells, NK cells, dendritic cells, and cytokine production have been described [5, 6]. 

A better understanding of the biology of the disease and tumor microenvironment has opened new ways for the development of immunotherapy-based treatments. The use of immunotherapy is of particular interest in this disease because the alteration of the immune system is further aggravated by the use of chemotherapeutic agents such as fludarabine and cyclophosphamide with rituximab (FCR) which are the current standards in frontline therapy. It is interesting to highlight that in a high percentage of patients the cause of death is related to immunodeficiency. Presumably, the activation of the immune system may ameliorate the immunodeficiency and repair the antileukemic immunity producing durable clinical responses.

## 2. Mechanism of Action of Lenalidomide

Lenalidomide is an antineoplastic agent that exerts its antitumor action through various mechanisms such as activation of the immune system, inhibition of angiogenesis, and direct antineoplastic effects. The mechanisms of action may vary according to the disease, but there is growing evidence indicating that lenalidomide does not have a direct cytotoxic effect on CLL cells, but instead, it acts primarily by promoting and restoring the function of the immune system. Contrarily, changes in the serum concentrations of VEGF or in the density of the microvasculature in the bone of CLL patients who responded to lenalidomide treatment have not been found [7].

Functional immune reconstitution seems essential for the antileukemic activity of lenalidomide in CLL [8]. When lenalidomide is administered in cycles of 21 days, there is a rapid increase of the number of lymphocytes in the off-treatment week [9]. The stimulation of the immune system seems to be pleiotropic affecting different cells and functions. Lenalidomide causes an overexpression of costimulatory molecules in leukemic lymphocytes inducing an “activation phenotype” that restores the humoral immunity and the production of immunoglobulins [10]. It also improves the function of T cells and the ability of leukemic cells to form synapses with T lymphocytes [11]. There is also an increase of the number and the cytotoxic capacity of NK cells and a reduction of the number and suppressor activity of Treg cells [12].

### 2.1. Effects of Lenalidomide on Leukemic Cells

In contrast to normal B cells, leukemic cells are poor antigen presenting cells. This is due to the fact that leukemic cells have a reduced expression of costimulatory molecules such as CD80 and CD86 and they have a defect in the formation of immunological synapse with T cells. After lenalidomide treatment, there is an overexpression of costimulatory molecules and activation markers in leukemic B cells such as CD40, CD80, CD86, CD54, CD95 (Fas), DR5 and HLA-DR [9, 13]. Immune activation in CLL and the overexpression of costimulatory molecules may not only be responsible for the antineoplastic activity of lenalidomide, but also for the tumor flare syndrome (TFR) that affects some CLL-treated patients [7]. It has also been observed that lenalidomide restores the humoral immunity, since it induces the expression of CD154 (also known as CD40L) on T cells, which not only increases the sensitivity of leukemic cells to apoptosis, but it also activates normal B cells favoring the production of antibodies. Among them, there are some anti-tumor antibodies such as anti-ROR1 [14].

### 2.2. Lenalidomide Activates Other Lymphocyte Subpopulations

 In CLL patients receiving lenalidomide as a first-line monotherapy, quantitative changes in lymphocyte subpopulations were observed after 21 days of treatment. There was a decrease in the number of CD19/CD5+ leukemic cells and an increase in the percentage of CD4 T cells, CD8 T cells, and NK cells [13]. It is worth mentioning that lenalidomide activates CD8 T cells and NK cells that play a key role in the tumor surveillance.

Using lenalidomide monotherapy as first line, it was reported that the absolute number of lymphocytes decreased (mainly at the expense of leukemic lymphocytes) after three cycles of treatment. There was an increase of IFN-*γ* produced by CD8 T cells and Treg compared with their levels before the treatment [15]. The early increase of IFN-*γ* produced by CD8 T cells indicates that the cytotoxicity mediated by these cells may be involved in the mechanism of action of lenalidomide [15]. Nevertheless, the levels of T cells returned to normal values after 14 months of treatment. Similarly, there was an increase in the levels of CD4 T cells that produce IL-2, IFN-*γ*, and TNF-*α* with respect to the baseline values and normal individuals. The elevation of CD4 T cells persisted for three months, returning to normal values at fourteen months [15]. 

Many of the immunomodulatory effects of lenalidomide occur via secretion of cytokines. In the study Ferrajoli et al., patients who respond to lenalidomide showed significant increases in the IL-2R, IL-6, and IL-10 levels [7] that may have a direct effect on immune cells. Likewise, Davies et al. demonstrated that lenalidomide is able (*in vitro*) to stimulate mononuclear cells in an IL-2-dependent manner, so they are able to kill myeloma cells [16]. This effect was abolished by the depletion of CD56+ cells suggesting that NK or NKT cells mediate this effect. Moreover, an increase of CD56+ cells in responding patients was observed, suggesting that the lenalidomide therapeutic activity may be mediated by the increase of the number and function of NK cells [16].

*In vitro*, lenalidomide stimulates NK cell activity at least through the production of IL-2 by T cells increasing the antibody-dependent cellular cytotoxicity (ADCC) mediated by these cells [17]. *In vivo*, an increase of NK cells in lenalidomide-treated patients has been observed; however, these cells showed a decrease in its activation level, having a concomitant enhancement of the cytotoxicity of CD8 T lymphocytes [18]. In addition to NK cells, lenalidomide also induces NKT cell expansion and stimulates its antitumor activity [19]. 

Overall, lenalidomide has a pleiotropic effect promoting several elements of the antitumor immune response. The role of each mechanism of action on the therapeutic activity of lenalidomide remains to be elucidated.

### 2.3. Mechanism of Action of Lenalidomide Associated with Rituximab

We have observed that in CLL, lenalidomide mainly acts promoting the proliferation and activation of NK cells *ex vivo*. However, the capacity of lenalidomide to promote the antileukemic activity of NK cells is limited. This is due to the fact that tumor cells are detected by NK cells through changes in their receptors ligand expression. However, leukemic cells of CLL patients express low levels of ligands of NK cell activating receptors, probably due to immune evasion mechanisms, being highly resistant to NK cell-mediated activation. To increase the cytotoxic activity against leukemia cells, it is necessary to favor the recognition of leukemia cells by NK cells. According to this idea, lenalidomide is an attractive agent for combination with rituximab. Rituximab allows the elimination of CD20+ leukemia cells through the receptor for IgG called CD16 from NK cells, by antibody-dependent cytotoxicity or ADCC. *In vitro,* it has been reported that lenalidomide decreases the leukemic cells CD20 expression, antagonizing the effect of rituximab [20], but this has not been confirmed [13]. In addition, we have found that the lenalidomide effect on CD20 expression on leukemic cells is variable, but independently, lenalidomide has a synergistic effect with rituximab. Following this idea, lenalidomide is an attractive agent to combine with other agents which favour the activity of NK cells.

## 3. Lenalidomide Efficacy

### 3.1. Use of Lenalidomide in Chronic Lymphocytic Leukemia

Lenalidomide, a derivative of thalidomide, is an immunomodulatory drug with significant activity in CLL. The efficacy of lenalidomide in monotherapy is comparable to other single cytotoxic agents used in this disease. In recent years, several phase II clinical trials have demonstrated the effectiveness of lenalidomide in CLL patients who relapsed or were refractory to previous treatments that included fludarabine; better results have been obtained when used as a first-line treatment [7, 9, 21]. 

The first clinical trial demonstrating the clinical activity of lenalidomide in CLL is a randomized phase II study analysing 45 patients with relapsed or refractory disease [21]. In this study, lenalidomide was initiated at 25 mg daily in 28 day cycles. After two cases of TLS, the starting dose was reduced to 5 mg, with escalations to a maximum of 25 mg. In heavily pretreated patients with CLL (51% fludarabine refractory), ORR was 47% and complete remission (CR) was 9% [21]. In another phase II trial, in 44 patients using a more cautious dosing schedule (starting at 10 mg daily and increasing 5 mg every 28 days to a maximum of 25 mg), overall response decreased to 32% (CR 7%), but side effects were significantly reduced [7]. In a phase I study using a low starting dose of 2.5 mg, and escalating 5 mg each 28 days to 10, 15, and 20 mg, one-third of patients could not escalate beyond 2.5 mg, although those who reached 20 mg did not suffer any dose-limiting toxicities [22]. This schedule with a low initial dose and further escalation has been adopted in subsequent clinical trials. In another study with a pulse dosing schedule of 21 days with 21 days off, global responses decreased to 16%, but toxicity was similar [10].

Of considerable interest is its usefulness in patients with adverse features. It has been reported that in patients with high-risk cytogenetic features, the ORR was 31–38%; in cases with nonmutated IgH, it was 24%; and in patients with fludarabine-refractory disease, it was 25–30% [7, 23]. Likewise, lenalidomide treatment, alone or in combination, became an effective alternative in these patients. In CLL patients with del(17p) pretreated with different regimens, 8 who were treated with lenalidomide obtained an ORR of 38% (CR 13%) and an overall survival (OS) of 11 months, which are better results than obtained with other combinations currently used in these patients [24]. The ORR improved up to 72% with lenalidomide monotherapy as first-line treatment with a prolonged followup of 47 months [25]. 

The overall response rate (ORR) of lenalidomide monotherapy as first-line therapy was 65% in elderly patients [26]. This study demonstrates that longer therapy and higher doses of lenalidomide are more effective to obtain responses. Nevertheless, the ORIGIN trial (NCT00910910), which has recently evaluated the use of lenalidomide treatment as an initial therapy for CLL patients of 65 or older, showed higher rates of death in patients treated with lenalidomide compared with those treated with chlorambucil (hazard ratio (HR) of 1.92). FDA has halted this study after determining that this treatment was unlikely to achieve an improved progression-free survival in these older patients (primary objective).

Table 1 shows the response, survival rates, and adverse effects of several studies using different regimens of lenalidomide monotherapy as first-line or salvage treatment in relapsed CLL patients. Most of these trials begin with a low dose and then try to escalate to the target dose [22, 26–28].

### 3.2. Lenalidomide with Rituximab in Induction

The efficacy of lenalidomide may be increased with the addition of other agents such as rituximab. In several phase II clinical trials, it has been demonstrated that the addition of rituximab improved the response rates without increasing the toxicity. In a high percentage of patients, the number of leukemic cells declines within 8 days after the start of the treatment.

Badoux et al. reported that with this combination, relapsed or refractory CLL patients achieved an ORR of 66% (including 12% CR) and an estimated 36-month survival of 71% [29]. In this study, lenalidomide was started with a continuous dose of 10 mg on day 9 of cycle 1 and rituximab was added weekly (375 mg/m^2^) during the first cycle and then monthly (cycles 3 to 12). Although it cannot be directly compared with the outcomes obtained in the monotherapy studies, the encouraging ORR and sustained responses observed suggest an increasing benefit with the addition of rituximab, with less side effects and better tolerance. Furthermore, rituximab administered before lenalidomide could also act as a debulking agent reducing the rate and severity of TFR. 

In another phase II clinical trial using lenalidomide and rituximab as the first-line therapy, overall responses were higher than 90%. Moreover, this combination was safe with an acceptable toxicity profile. Even seven patients with (17p) deletion showed an ORR of 53% (CR 13%) [30]. Responses with this combination were also obtained in 71% of patients refractory to lenalidomide monotherapy [31]. 

The overall conclusion from these studies is that continuous treatment with this combination may probably provide accumulative benefits [32]. Thus, combination of lenalidomide with rituximab could offer an effective alternative for patients who relapsed after fludarabine-containing chemoimmunotherapy. Nevertheless, further studies must be implemented to obtain definite conclusions.

### 3.3. Other Combinations with Lenalidomide Induction

A current phase II study using lenalidomide in combination with ofatumumab demonstrates an acceptable toxicity profile [33], and similar outcomes are shown in several phase I trials with other combinations (fludarabine, cyclophosphamide, and lenalidomide [34]; fludarabine, rituximab, and lenalidomide [35]; the same combination followed of maintenance with lenalidomide and rituximab [36] or in combination with bendamustine [37] or flavopiridol [38] or alemtuzumab). The tolerated dose of lenalidomide in these combinations is generally low (5–10 mg) and its efficacy is higher than in lenalidomide monotherapy. In fludarebine-based combinations even at the lowest dose level, dose-limiting toxicities occurred in most patients.

The preliminary results obtained from a phase II study with lenalidomide plus dexamethasone show a significant activity in previously untreated CLL patients. Moreover, it is generally well tolerated and reduces the incidence of side effects, such as TFR, enabling the escalation to higher dose of lenalidomide [39].

In another trial using lenalidomide consolidation after 6 cycles of pentostatin, cyclophosphamide, and rituximab (PCR-L), an increase in the response quality was observed and negative minimal residual disease (MRD) was shown in some cases. The treatment-free survival (TLS) was higher than a historical control (79% versus 66% at 30 months) and the main toxicity observed was hematologic, but no cases of TLS or TFR were described [40]. An improved quality of the responses was also reported using lenalidomide as consolidation after FR or FCR treatment [41, 42]. Currently, lenalidomide is under evaluation for maintenance “Continuum Studio” and in patients with monoclonal B lymphocytosis.

Table 2 shows the different studies using lenalidomide in combination with other agents in CLL patients.

## 4. Dose and Schedule of Lenalidomide in Patients with CLL

The 25 mg dose used in early studies was associated with excessive toxicity, and then subsequent trials started with lower doses (2.5–5 mg) progressively increased to 10–25 mg. In the majority of studies, the median of the tolerated dose was 10 mg. The use of lower doses may be associated with lower response rates, and dose escalations must be implemented to improve efficacy. Currently, daily doses used in trials range between 2.5 and 10 mg.

One of the open issues that needs further investigation is why a continuous dosing is more effective than 21 days schedules with a week off. The “recovery” of peripheral lymphocytosis observed using intermittent dosing of lenalidomide led to the current continuous daily dosing [9], since lymphocytosis recovery has not been noted in this regimen. Continuous dosing may prevent the leukemia cells recovery, inhibit the production of cytokines that promote the leukemia cells survival, and may also prevent the support of malignant cells by stromal cells [7]. Likewise, in patients with adverse prognostic features who were heavily pretreated and received 21 days of treatment with other 21 days off, the decrease of the toxicity, but also the efficacy, was observed [10].

As of yet, none of the clinical characteristics analyzed have allowed us to predict the response to lenalidomide treatment. Only one study reported that responders had a higher number of neutrophils than nonresponders [9].

## 5. Side Effects

The most common grade 3-4 adverse events of lenalidomide treatment were neutropenia, thrombocytopenia, and anemia. Tumor lysis syndrome (TLS), tumor flare reaction (TFR), and venous thromboembolism (VTE) will be discussed later. Skin rash, elevated liver enzymes, and phosphorus (P) and calcium (Ca) alterations are even more uncommon.

### 5.1. Tumor Flare Reaction (TFR)

“Tumor flare reaction” (TFR) is a side effect unique of lenalidomide treatment in this disease that consists of the appearance of an increase in swelling of lymph nodes, spleen, and liver, with or without fever, erythema usually associated with local or generalized rash (maculopapular, erythematous, and nonpruritic), bone pain, and an increase in the number of lymphocytes. It is a self-limited and transient effect and can be managed with NSAIDs (ibuprofen 400–600 mg/6 h) or a short course of steroids in severe cases. It is important to recognize it to avoid confusion with disease progression and improperly discontinue the treatment.

Using a starting dose of 25 mg, lenalidomide induced a significant response in CLL patients, associated with tumor lysis syndrome and TFR in a high percentage of cases [21]. These toxicities have been associated with a high starting dose and a rapid escalation of lenalidomide. With high doses of lenalidomide, it occurs within 6 days of treatment [7]; it is more common in the first few cycles of therapy and in previously untreated patients with a more robust immune system [9]. Although TFR is most common during the first cycle, repeated flare symptoms were also noted in 16% of the cycles upon resuming the lenalidomide treatment after the week off of each cycle, and it was observed as late as in cycle 28 [25]. It is also more common in patients with advanced stages of the disease and in patients with lymph nodes larger than 5 cm [7]. Contrarily, it has not been described in cases with low tumor mass, where lenalidomide is used as consolidation.

TFR was significantly reduced using low starting doses and using slower dose escalations [7]. In the study reported by Chanan Khan, using 20 mg of prednisone in the first 5 days and 10 mg another 5 days as prophylaxis, there was a decreasing in severity, but not in TFR incidence; nevertheless, none of the patients had to stop the treatment or reduce the dose. In this study, the onset of the syndrome was unrelated to the previous lymphocyte count or burden of the disease [21]. In a clinical trial reported by Chen et al., almost one-third of patients were treated with lower doses of prednisone (25–50 mg for 5–10 days) and TFR was common, but mild [9].

The frequency of TFR also appears to be lower when lenalidomide is used in combination with rituximab. Using these drugs, TFR was also milder (grades 1 and 2) and occured during the first cycle of treatment [30]. The use of corticosteroids with lenalidomide plus dexamethasone is very interesting since it has significant activity in previously untreated CLL, is generally well tolerated and reduces dramatically the incidence of TFR symptoms. However, the use of other drugs with lenalidomide requires careful consideration of dosing and scheduling; for example, sequential treatment with ofatumumab and lenalidomide may be associated with a higher rate of TFR (57%) than shown with concomitant therapy [46]. 

It has been suggested that TFR, a side effect observed only in CLL, may be secondary to the immune system activation. Accordingly, it has been reported that it is highly correlated with an overexpression of costimulatory molecules CD40, CD80, and CD86; and consequently, this “flare” may be due to an increased ability of leukemic B cells to present antigens and inducing an antitumoral response [47]. Accordingly, TFR has been correlated with clinical response [48]; however, this association has not been corroborated in all studies. On the other hand, it has also shown that progression-free survival in TFR patients is not better [23, 49].

The development of TFR has also been reported as a predictor of response [50]; however, it has not been corroborated in all studies.

### 5.2. Tumor Lysis Syndrome (TLS)

Tumor lysis syndrome (TLS) is a specific adverse event more common in patients with high tumor burden or with regimens with a high starting dose of lenalidomide. TLS is a group of metabolic complications that appears after the lenalidomide treatment caused by the release of breakdown products by the lysis of leucemic cells. This syndrome includes hyperkalemia, hyperphosphatemia, hyperuricemia, hyperuricosuria, and hypocalcemia. It is mainly observed in the first 15 days of the treatment and it is more common in patients with bulky disease, moderate renal impairment, and elevated baseline uric acid levels. In some cases, it may progress to renal failure or arrhythmias that can be fatal [51].

Allopurinol (300 mg) is used for the prophylaxis of TLS, starting 3 days before the starting of the treatment and it is also used at the first cycle of each dose escalation (Celgene Corporation 2010). The dose should be adjusted in patients with renal insufficiency and patients should be adequately hydrated, drinking 8–10 glasses of water (240 mL each glass) a day for 14 days in the first cycle and for 14 days after each dose escalation (always taking into account the patient's cardiovascular status and the possibility of overloading volume). Additionally, uric acid, P, Ca, and creatinine levels must be monitored; and patients with previous renal failure requiring dialysis or having creatinine clearance lower than 60 mL/min should be excluded.

### 5.3. Venous Thromboembolism (VTE)

Venous thromboembolism occurs in 5% of patients at 2–4 months after the starting lenalidomide treatment and can be avoided with appropriate prophylaxis. It has been suggested that lenalidomide may increase the risk of VTE due to endothelial cell damage caused by the presence of high TNF-*α* serum levels; aspirin may be useful in its prophylaxis [50]. Celgene has updated the prophylactic anticoagulation protocols in patients with CLL; these guidelines include the use of aspirin in patients with 0-1 risk factors for VTE and low molecular weight heparin (40 mg of enoxaparin or equivalent) or oral anticoagulants (INR target of 2-3) in patients with 2 or more thrombosis risk factors.

## 6. Conclusions

Lenalidomide alone or in combination (mainly with rituximab) offered an effective therapeutic alternative for patients who relapsed after fludarabine-containing chemoimmunotherapy. Consequently, it is necessary to prospectively compare this combination with other commonly used salvage regimens. We should pay special attention to TFR and TLS in early cycles of treatment and it is important to recognize them to avoid confusion with disease progression and improperly suspend the treatment. Although clinical responses to therapy occurred early in this treatment regimen, patients who received a long-term therapy obtained an improvement in the quality of the response. Durable responses may be achieved, although it needs a long time and the treatment must be prolonged until progression and/or CR. Shorter treatments can be effective when lenalidomide is used in combination with other drugs. Combination with agents that act synergistically favouring the activity of the immune system and agents which do not produce myelosuppression is of particular interest.

## Figures and Tables

**Table 1:** Lenalidomide monotherapy trials in patients with CLL.

References	Regimen	No	Response	TLS	TFR	AE grade 3-4
Chanan Kh [21] 2007 Phase II Relapsed or refractory	Lenalidomide 5 mg per day escalating up to 25 mg per day	45	ORR 47% RC 9% OS: NR TFS: NR	5%	58%	Neutropenia 70% Thrombocytopenia 45% Anemia 18%

Ferrajoli [7] 2008 Phase II Relapsed or refractory	Lenalidomide 10 mg per day escalating up to 25 mg/day (5 mg each 28 days) Median 10 mg	44	ORR 32% RC: 7% OS 73% at 14 months TFS: NR	0	12%	Neutropenia 41% Thrombocytopenia 13% Anemia 3%

Chen [9, 25] 2011 Phase II Untreated	Lenalidomide 2.5 mg per week escalating up to 10 mg or 25 mg if no response Median 18 cycles (2–33)	25	ORR 72% OS 85.3% TFS 68.8% (estimated at 3 years)	0	88%	Neutropenia 76% Thrombocytopenia 28% Anemia 20%

Aue 2010 [10] Phase II Relapsed or refractory	Lenalidomide pulses: 10–20 mg for 21 days followed by 21 days of rest (4–8 cycles)	31	ORR 16% RC 0 PFS responding 16 m versus 6 m	0	53%	Neutropenia 56% Thrombocytopenia 30% Anemia 15% Infection

Badoux [26] 2011 Phase II Elderly Untreated	Lenalidomide 5 mg monthly increases to 25 mg as tolerated until disease progression or unacceptable toxicity	60 (>65 years) median 71 years	ORR 65% RC 10% OS 88% TFS 60% (estimated at 2 years)	0	52%	Neutropenia 83% Thrombocytopenia 47% Anemia <1% Infection 10%

Lamanna [27] Phase II Treated and untreated	Continuous low dose 2.5 mg to 5 mg is increased if progression (up to 20 mg)	21		7%	47%	Neutropenia 52% Thrombocytopenia 24%

Wendter [28] CLL-009 2011 Phase II Relapsed or refractory	Initial doses of 5, 10, or 15 mg escalating up to 25 mg (dose finding)	60	ORR 37% RC 3.4%	3.4%	10%	Neutropenia 41,7% Thrombocytopenia 25% Anemia 8.3%

Wendter [22] 2012 CLL-001 Phase II Treated	Continuous cycles of 28 days increasing from 2.5 mg to 25 mg	52	ORR 12% RC 0% SD 58%		10%	Neutropenia 65% Thrombocytopenia 33% Anemia 10%

TLS: tumor lysis syndrome; TFR: tumor flare reaction; AE: adverse effects; NR: not reported; ORR: overall response; CR: complete remission; OS: overall survival; SD: stable disease; TFS: treatment-free survival; m: months.

**Table 2:** Trials using lenalidomide in combination in CLL patients.

References	Regimen	No.	TLS %	TFR %	AE 3-4 (%)	Response
Chanan Khan [31] 2006-7 Phase II Relapsed or refractory	Lenalidomide 10 mg per day escalated up to 5 mg each 1-2 week (max 25 mg) 21 days each 28 Rituximab 375 mg/m^2^ days 1, 8, and 15 (cycle 1) and days 1 and 15 (cycles 2–6)	30	5	8	Neutropenia 70% Thrombocytopenia 45% Anemia 18%	ORR 57.7% RC 18% TFS 19.4 months ORR 38% (high risk cytogenetic)

James 2011 [30] Phase II frontline CLL Research Consortium CRC-014	Lenalidomide cycle 1: 2.5 mg; cycles 2–7: 5 mg escalated up to 10 mg (days 1–21 each 28) Rituximab: 50 mg/m^2^ day 29; 325 mg/m^2^ day 31; 375 mg/m^2^ day 33 (cycle 1), 375 mg/m^2^ weekly (cycle 2) and 375 mg/m^2^ day 1 (cycles 3–7)	69		1.4	Neutropenia 49% Thrombocytopenia 6% Anemia 11%	ORR 95% (<65 y) ORR 78% (>65 y) RC 20% (<65 y) RC 8% (>65 y) TFS 19-20 m

Badoux [29, 43] Phase II relapsed or refractory	Lenalidomide was started on day 9 at cycle 1 and on day 1 of the cycles 3–12: 10 mg continuously Rituximab 375 mg/m^2^ weekly during cycle 1 and on day 1 cycles 3 to 12	59	1.7	27	Neutropenia 73% Thrombocytopenia 34% Anemia 15% Infection 15%	ORR 66% RC 12% OS: 71% at 36 months TFS: 17.4 m

Veliz 2009 [32] Phase II Relapsed or progression after rituximab Heavily treated	Lenalidomide cycle 1: 2.5 mg (days 1–7), 5 mg (days 8–15), 10 mg (15–21 days) followed by 7 days of rest and then 20 mg 21 each 28 days Rituximab 375 mg/m^2^ weakly each 4 weeks (day 15)	10	(RF) 12	12	Neutropenia 41%	ORR 30% RC 0

Chen 2012 [39] Phase II frontline	Lenalidomide: 5 mg per day escalated 5 mg each 28 days (max 25 mg) Dexamethasone: 12 mg days 1–4; 14, 21, and 28 Maximum 18 cycles	18	0	5	Neutropenia 53% Neutropenia febrile 24% Thrombocytopenia 12%	0R 59% RC 1% RP 53%

Badoux [33] Phase II Relapsed or refractory	Lenalidomide 10 mg per day Ofatumumab weekly	16	NR	13	Neutropenia 50% Anemia 13%	ORR 63% RC: 13% OS: NR TFS: NR

Ferrajoli [44] 2012 Phase II Relapsed or refractory	Lenalidomide 10 mg day 9 continued for 24 months Ofatumumab weekly for 3 weeks (300 mg week; 1000 mg week 2 and thereafter) monthly (months 2–6); every other month (months 7–24)	36	0	0	Neutropenia 47% Thrombocytopenia 9% Anemia 6%	ORR 68% RC 24%

Blum [38] 2011 Phase I Relapsed or refractory very adverse	Lenalidomide 2.5 mg escalating up to 25 mg days 1–21 Flavopiridol 30 mg/m^2^ in bolus followed of 30–50 mg/m^2^ days 1, 8, and 15 (cycle 1) and then days 3, 10 and 17	30	3	7	Neutropenia 47% Thrombocytopenia 60% Anemia 33%	ORR 57% RC: 0% OS 7 m TFS 23 m

GIMEMA [34] LLC 606 Phase I Relapsed or refractory	Lenalidomide 2.5 mg escalating up to 15 mg Cyclophosphamide Fludarabine	9	0	11	Neutropenia transitory 3-4 in the majority of the patients	ORR 67% RC 33% NR NR

Egle [36] 2011 Phase I/II Frontline	Lenalidomide 2.5 mg/day (days 7–21) escalating up to 25 mg (day 1–21) Fludarabine 40 mg/m^2^ 1–3 Rituximab 375 m/m^2^ day 3 cycle 1; 500 mg/m^2^ day 1 cycles 2–6 Maintenance: R: 375 mg/m^2^ cycles 2, 4, and 6 Lenalidomide (maximum tolerated dose)	45	0	0	Neutropenia 88%	ORR 87% RC 49% NR NR

Flinn [45] 2012 Phase I-II Frontline	Rituximab 375 mg/m^2^ (cycle 1); 500 mg/m^2^ (cycle 2–6) Fludarabine 25 mg/m^2^ (days 1–3) Lenalidomide 2.5–5 mg (days 8–28) 6 cycles	51	6	0	Neutropenia 47% Anemia 14% Thrombocytopenia 6 Rash 14%	ORR: 78% RC 19% PFS 71% OS 88% (median FU 21 months)

Brown [35] Phase I Frontline	Fludarabine: 25 mg/m^2^ (days 3–5) Rituximab: 50 mg/m^2^ day 1 and 325 mg/m^2^ day 3 Lenalidomide: 2.5 mg to alternate days (21 each 28 days) Followed by two cycles of consolidation with lenalidomide	9	1/9	2/9	Neutropenia 66% Thrombocytopenia 2/9 Allergy Syndrome hand-foot	ORR 56% RC 1/9

TLS: tumor lysis syndrome; TFR: tumor flare reaction; AE: adverse effects; NR: not reported; ORR: overall response; CR: complete remission; OS: overall survival; TFS: treatment-free survival; y: year; m: month; RF: renal failure.
